# Genomic alterations at the basis of treatment resistance in metastatic breast cancer: clinical applications

**DOI:** 10.18632/oncotarget.25810

**Published:** 2018-08-03

**Authors:** Angela Toss, Federico Piacentini, Laura Cortesi, Lucia Artuso, Isabella Bernardis, Sandra Parenti, Elena Tenedini, Guido Ficarra, Antonino Maiorana, Anna Iannone, Claudia Omarini, Luca Moscetti, Massimo Cristofanilli, Massimo Federico, Enrico Tagliafico

**Affiliations:** ^1^ Department of Oncology and Haematology, Azienda Ospedaliero-Universitaria Policlinico di Modena, Modena, Italy; ^2^ Center for Genome Research, University of Modena and Reggio Emilia, Modena, Italy; ^3^ Department of Medical and Surgical Sciences, University of Modena and Reggio Emilia, Modena, Italy; ^4^ Department of Pathology, Azienda Ospedaliero-Universitaria Policlinico di Modena, Modena, Italy; ^5^ Department of Diagnostics, Clinical and Public Health Medicine, University of Modena and Reggio Emilia, Modena, Italy; ^6^ Department of Medicine-Hematology and Oncology, Robert H Lurie Comprehensive Cancer Center, Feinberg School of Medicine, Northwestern University, Chicago, IL, USA

**Keywords:** breast cancer, treatment resistance, molecular characterization, next-generation sequencing, somatic mutations

## Abstract

The standard of care for breast cancer has gradually evolved from empirical treatments based on clinical-pathological characteristics to the use of targeted approaches based on the molecular profile of the tumor. Consequently, an increasing number of molecularly targeted drugs have been developed. These drugs target specific alterations, called driver mutations, which confer a survival advantage to cancer cells. To date, the main challenge remains the identification of predictive biomarkers for the selection of the optimal treatment. On this basis, we evaluated a panel of 25 genes involved in the mechanisms of targeted treatment resistance, in 16 primary breast cancers and their matched recurrences, developed during treatment. Overall, we found a detection rate of mutations higher than that described in the literature. In particular, the most frequently mutated genes were ERBB2 and those involved in the PI3K/AKT/mTOR and the MAPK signaling pathways. The study revealed substantial discordances between primary tumors and metastases, stressing the need for analysis of metastatic tissues at recurrence. We observed that 85.7% of patients with an early-stage or locally advanced primary tumor showed at least one mutation in the primary tumor. This finding could explain the subsequent relapse and might therefore justify more targeted adjuvant treatments. Finally, the mutations detected in 50% of relapsed tissues could have guided subsequent treatment choices in a different way. This study demonstrates that mutation events may be present at diagnosis or arise during cancer treatment. As a result, profiling primary and metastatic tumor tissues may be a major step in defining optimal treatments.

## INTRODUCTION

Breast cancer (BC) is a heterogeneous disease that develops and progresses from alterations in the genes that govern cell growth, proliferation and differentiation [1, 2]. At the same time, these molecular alterations are responsible for primary and secondary treatment resistance and may represent a major limitation to cancer treatment efficacy. In the last two decades, increasing knowledge of genomic abnormalities associated with gain of function or downstream signal activation involved in BC evolution allowed for new therapeutic approaches “tailored” on identified molecular alterations. The revolutionary era of targeted therapy shifted the classic paradigm of BC treatment from an approach based on pathological and clinical characteristics [3] to personalized medicine. This is based on the match between molecular alteration conferring a survival advantage to cancer cells, and the targeted drug [4]. An increasing number of molecularly targeted drugs are currently available for clinical practice or in the context of clinical trials. Nowadays, the main challenge remains the identification of predictive biomarkers for the selection of optimal treatment, in order to spare patients from treatment-associated side effects and to minimize the overall cost [5]. On this basis, parallel to the development of new therapeutic strategies, researches are looking for molecular biomarkers able to predict response to those treatments. For some of these targeted therapies, predictive biomarkers have already been identified in clinical trials [6]. In particular, *PIK3CA* mutations have already been shown to predict sensitivity to Everolimus [7], Buparlisib [8] and Taselisib [9] as well as resistance to Lapatinib [10]. *AKT1* mutations can predict sensitivity to Everolimus [7], while *ESR1* mutations can predict sensitivity to Fulvestrant and resistance to Exemestane [11]. Furthermore, HER2-negative patients with an *ERBB2* somatic mutation are potentially good candidates for HER2-targeted therapy [12, 13], as already shown by Neratinib in two recently published clinical trials [14, 15].

The main purpose of our study was to investigate the mechanisms of treatment resistance in a sample of metastatic BC patients. Since BC behaves as an evolving entity, with metastases acquiring different biological profiles as compared to their matched primary tumors [16, 17], we evaluated a panel of 25 genes involved in the mechanisms of endocrine and targeted treatment resistance in paired BC samples (primary and recurrence) of patients with metastatic BC.

## RESULTS

### Patient and sample characteristics

The tumor characteristics of the 16 patients enrolled in the study are described in Table 1. The median age at BC diagnosis was 57 years (range 35–81). Following the classification proposed in the 13th St Gallen International Breast Cancer Conference in 2013, seven patients (43.75%) were diagnosed with Luminal A-like BC. This means estrogen-receptor positive, progesterone-receptor ≥20%, HER2 negative, and low levels of MIB1 (<20%). The other 9 patients (56.25%) had Luminal B-like BC (estrogen-receptor positive and progesterone-receptor <20% or HER2 positive or levels of MIB1 ≥20%). Of these patients, 4 were Luminal B-like HER2 positive.

**Table 1:** Patients and sample characteristics

Patient number	Age at diagnosis	Primary tumor molecular subtype	Primary tumor characteristics	TNM	Stage at diagnosis	Sites of relapse	Biopsy of the relapse	Relapse characterisitics
Patient 1	56	LUMINAL A-like	CDI G3, ER 50%, PR 70%, MIB1 20%, HER2 negative	pT2pN1(3/17)M0	**IIB**	SKIN (Local Relapse)	SKIN NODULE EXCISION	ER 100%, PR 20%, HER2 negative
Patient 2	35	LUMINAL B-like (HER2+)	CDI G3, ER 60%, PR 2%, MIB1 ?, HER2 positive	pT2(25mm)pN0(0/24)M0	**IIA**	LUNG	SINGLE LUNG NODULE RESECTION	ER 95%, PR 95%, HER2 positive
Patient 3	45	LUMINAL B-like	CLI G3, ER 90%, PR 90%, MIB1 50%, HER2 negative	pT2(multif)pN1(1/15)M0	**IIB**	SKIN (Local Relapse)	SKIN PUNCH	ER 90%, PR 60%, HER2 negative
Patient 4	56	LUMINAL A-like	CDI G2, ER 95%, PR 95%, MIB1 8%, HER2 negative	pT2pN3(11/13)M1	**IV**	BONE, SKIN (Scalp)	SKIN PUNCH	ER 90%, PR 50%, MIB 20%, HER2 negative
Patient 5	52	LUMINAL A-like	CDI G2, ER 90%, PR 80%, MIB1 15%, HER2 negative	pT1cpN1(1/19)M0	**IIA**	IPSILATERAL AXILLARY LYMPH NODES, BONE, LIVER	IPSILATERAL AXILLARY LYMPH NODES	ER 10%, PR 1%, HER2 negative
Patient 6	71	LUMINAL B-like (HER2+)	CDI G3, ER 100%, PR 10%, MIB1 15%, HER2 positive	pT1cpN0M0	**IA**	IPSILATERAL SUPRACLAVICULAR LYMPH NODES	IPSILATERAL SUPRACLAVICULAR LYMPH NODES	ER 90%, PR 1%, HER2 positive
Patient 7	57	LUMINAL A-like	CDI G3, ER 90%, PR 40%, MIB1 8%, HER2 negative	pT2,pN3(17/33),M1	**IV**	CONTRALATERAL AXILLARY LYMPH NODES, BONE	CONTRALATERAL AXILLARY LYMPH NODES	ER 0%, PR 0%, HER2 negative
Patient 8	47	LUMINAL A-like	CDI G3, ER 85%, PR 9%, MIB1 16%, HER2 negative	pT1cpN1(2/20)M0	**IIA**	CONTRALATERAL AXILLARY LYMPH NODES	CONTRALATERAL AXILLARY LYMPH NODES	ER 100%, PR 100%, HER2 negative
Patient 9	73	LUMINAL A-like	CDI G2, ER 100%, PR 90%, MIB1 15%, HER2 negative	pT1cpN0M0	**IA**	BONE, LUNG, LIVER, LYMPH NODES	LIVER	ER 85%, PgR 0%, MIB1 10%, HER2 negative
Patient 10	71	LUMINAL B-like (HER2+)	CDI G3, ER 15%, PR neg, MIB1 10%, HER2 positive	pT2,pN3(39/39),M0	**IIIC**	LYMPH NODES, IPSILATERAL CHEST WALL	IPSILATERAL CHEST WALL	ER neg, PR neg, HER2 positive
Patient 11	56	LUMINAL B-like	CDI G2, ER 98%, PR 0%, MIB1 25%, HER2 negative	pT1c(multif),pN0,M0	**IA**	IPSILATERAL AXILLARY LYMPH NODES	IPSILATERAL AXILLARY LYMPH NODES	ER 15%, PR neg, MIB1 40%, HER2 negative
Patient 12	81	LUMINAL B-like	CLI G3, ER 80%, PR <1%, MIB1 15%, HER2 negative	cT3,cN+,M0	**IIIA**	BREAST, LYMPH NODES, BONE, LIVER	IPSILATERAL MASTECTOMY	ER neg, PR neg, MIB1 40%, HER2 negative
Patient 13	61	LUMINAL B-like	CLI G3, ER 90%, PR 90%, MIB1 70%, HER2 negative	pT2,pN0(0/19),M0	**IIA**	BREAST, IPSILATERAL AXILLARY SOFT TISSUES	IPSILATERAL AXILLARY SOFT TISSUES	ER 50%, PR neg, MIB1 25%, HER2 positive
Patient 14	48	LUMINAL B-like	CDI G3, ER 95%, PR 95%, MIB1 80%, HER2 negative	cT3,cN+,M0	**IIIA**	BONE, LUNG, LIVER, LYMPH NODES	LIVER	ER 95%, PR 3%, MIB1 30%, HER2 negative
Patient 15	53	LUMINAL B-like (HER2+)	CDI G3, ER 90%, PR 30%, MIB1 10%, HER2 positive	pT1c,pN2(6/16),M0	**IIIA**	IPSILATERAL BREAST (CUTANEOUS)	IPSILATERAL BREAST SKIN PUNCH	ER 90%, PR neg, MIB1 25%, HER2 positive
Patient 16	45	LUMINAL B-like	CDI G3, ER 90%, PR 1%, MIB1 40%, HER2 negative	IBC cT4cN+M0 (right)	**IIIB**	CONTRALATERAL BREAST, RIGHT CHEST WALL, BONE	RIGHT CHEST WALL	ER 30%, PR neg, MIB1 20%, HER2 negative

Discordant ER, PR and HER2 reported in red.

At presentation, 3 patients (18.75%) had Stage IA BC, 4 patients (25%) had Stage IIA, 2 patients (12.5%) had Stage IIB, 3 (18.75%) had Stage IIIA, 1 (6.25%) had Stage IIIB, 1 (6.25%) had Stage IIIC and 2 (12.5%) had Stage IV disease. The most common sites of relapse were lymph-nodes (9 patients, 56.25%), bones (7 patients, 43.75%) and skin (6 patients, 37.5%). As regards the biopsy of the relapse/metastasis, 7 patients (43.75%) underwent skin or soft tissues biopsy, 5 patients (31.25%) had lymph-node biopsy, 2 patients (12.5%) had liver biopsy, one patient (6.25%) underwent single lung metastasis resection, and lastly one patient (6.25%) underwent ipsilateral mastectomy for a local recurrence. In 8 cases (50%), the relapse/metastasis maintained the same immunohistochemical characteristics [i.e. the same hormone receptors (HR) and HER2 status] of the primary tumor, whereas 8 (50%) changed during progression. More specifically, estrogen receptor (ER) and progesteron receptor (PR) were considered positive when ≥1%, according to the last ASCO/CAPS Guidelines [18]. Rates of discordance were 18.75%, 31.25% and 6.25% for ER, PR and HER2, respectively.

### Predictive value of primary tumor mutations

Two patients (12.5%) received neoadjuvant chemotherapy (anthracycline and taxane-based), whereas 8 patients (50%) received adjuvant chemotherapy (1 anthracycline and taxane-based, 3 anthracycline-based, 2 taxane-based and 2 patients with cyclophosphamide, methotrexate and fluorouracil). One patient (Patient 12) received endocrine treatment (letrozole and fulvestrant) with neoadjuvant purposes, but she achieved no response and underwent radical mastectomy with axillary lymph node dissection. All primary tumor tissues analyzed were taken before initiation of systemic treatment. Six patients (37.5%) received adjuvant tamoxifen alone or with LHRH analogues, 3 patients (18.75%) received adjuvant aromatase inhibitors alone or with LHRH analogues, whereas 4 patients (25%) received an adjuvant sequence of tamoxifen and aromatase inhibitors with or without LHRH analogues. None of the patients, including those with HER2-positive tumor, received HER2-targeted therapy in the neoadjuvant or adjuvant setting.

Two patients (12.5%) were diagnosed with *de novo* Stage IV BC, whereas the other 14 patients (87.5%) developed loco-regional or distant recurrence after receiving treatment for primary BC and particularly during neo/adjuvant endocrine therapies. The median disease-free survival (DFS) time for these patients was 35.38 months (range 3–58) from surgery or from the end of adjuvant chemotherapy. In more details, 4 patients (25%) had primary resistance to endocrine therapy, i.e. they relapsed while on the first 2 years of adjuvant endocrine therapy, or progressed within the first 6 months of first-line endocrine therapy. On the other hand, the other 12 patients (75%) developed secondary endocrine resistance.

**Figure 1:**
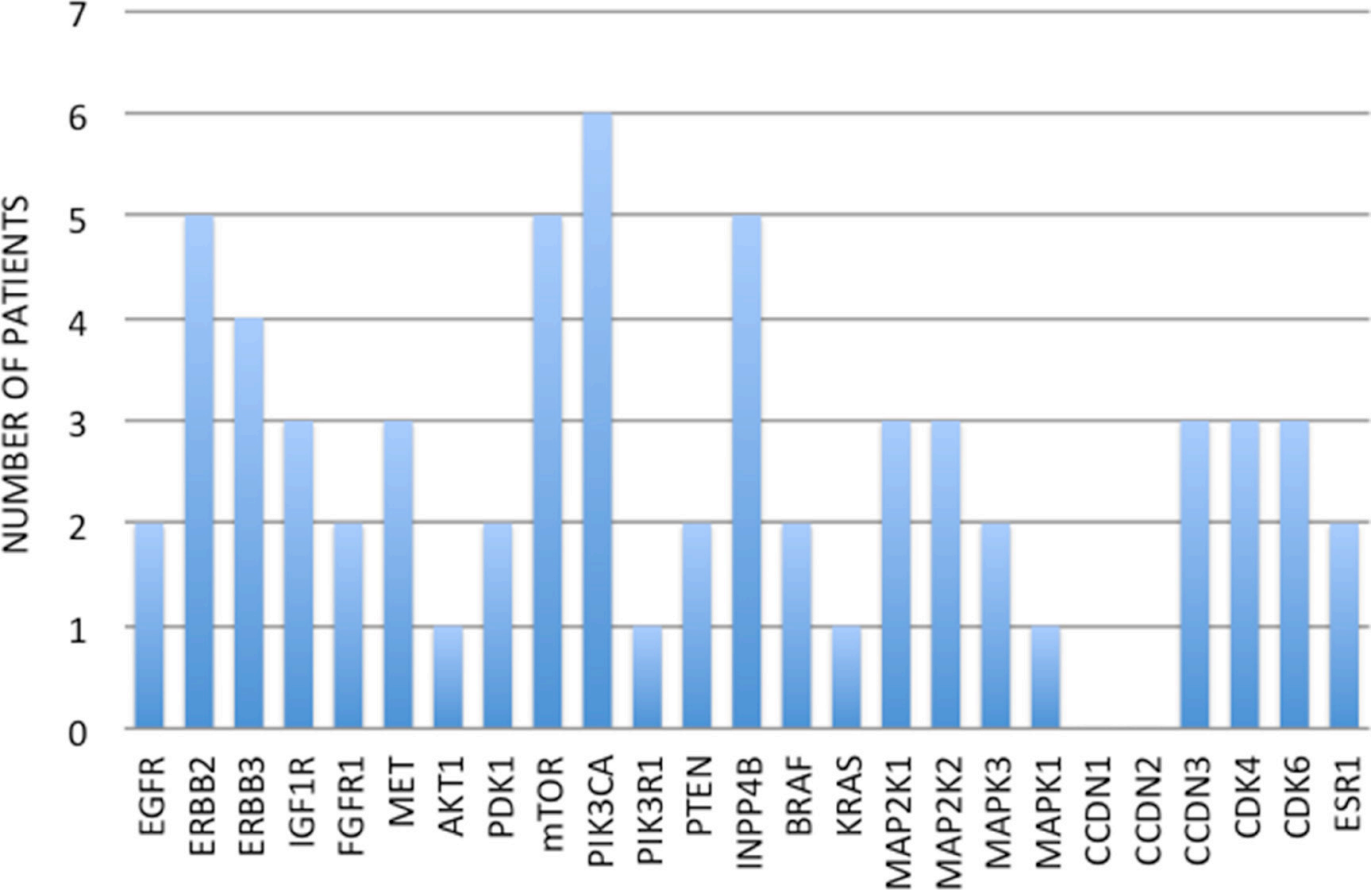
Number of patients for each primary tumor mutation

Among the 16 patients, 32 breast tumors were profiled, including 16 primary breast tumors, 10 loco-regional recurrences (ipsilateral breast, chest wall or supraclavicular/axillary lymph nodes) and 6 distant metastases (skin, contralateral axillary lymph nodes, liver and lung). Fourteen patients (87.5%) showed at least one mutation in one of the 25 genes involved in the mechanisms of targeted treatment resistance. Five patients (31.25%) had only one mutated gene, 2 patients (12.5%) had two mutated genes, whereas the other 7 patients (50%) had three or more mutated genes. Overall, we found 64 mutated genes in 16 primary tumors (Figure 1). The most common mutation detected in primary tumor tissues was *PIK3CA* (6 patients, 37.5%), followed by *ERBB2, mTOR* and *INPP4B* (5 patients for each one, 31.25%) (Figure 1).

### Predictive value of relapse mutations

Among the 16 patients, 10 developed loco-regional recurrences in the previously operated breast or chest wall or in the ipsilateral supraclavicular/axillary lymph nodes, whereas 10 patients developed distant metastases (bone, skin, lymph nodes, liver and lung). As far as tissue samples are concerned, 10 loco-regional recurrences and 6 distant metastases have been profiled. More precisely, 10 patients have been biopsied on the site of the first relapse during adjuvant treatments, while 5 patients were biopsied on a metastatic site in progression under treatment in the metastatic setting.

**Figure 2:**
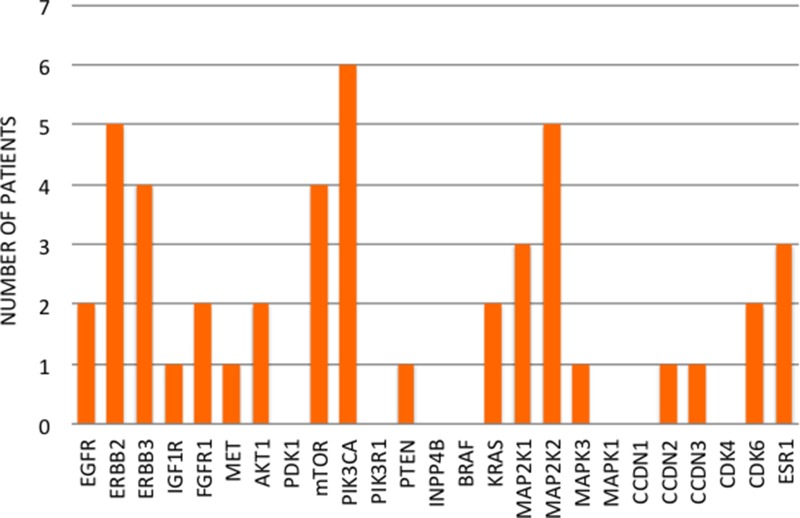
Number of patients for each relapse mutation

Two patients (12.5%) received only one line of therapy in the metastatic setting, while 3 patients (18.75%) received two lines of therapy, and the other 11 patients (68.75%) received three or more lines of therapy in the metastatic setting. Thirteen patients (81.25%) showed at least one mutation in one of the 25 genes involved in the mechanisms of targeted treatment resistance. Five patients (31.25%) had only one mutated gene and 2 patients (12.5%) had two mutated genes, whereas the other 6 patients (46.15%) had three or more mutated genes. Overall, we found 46 mutated genes in 16 local or distant relapses (Figure 2). The most common mutation detected in relapsed tissues was *PIK3CA* (6 patients, 37.5%), followed by *ERBB2* and *MAP2K2* (5 patients for each, 31.25%) (Figure 2).

### Comparison between primary tumor and relapse mutations

**Figure 3:**
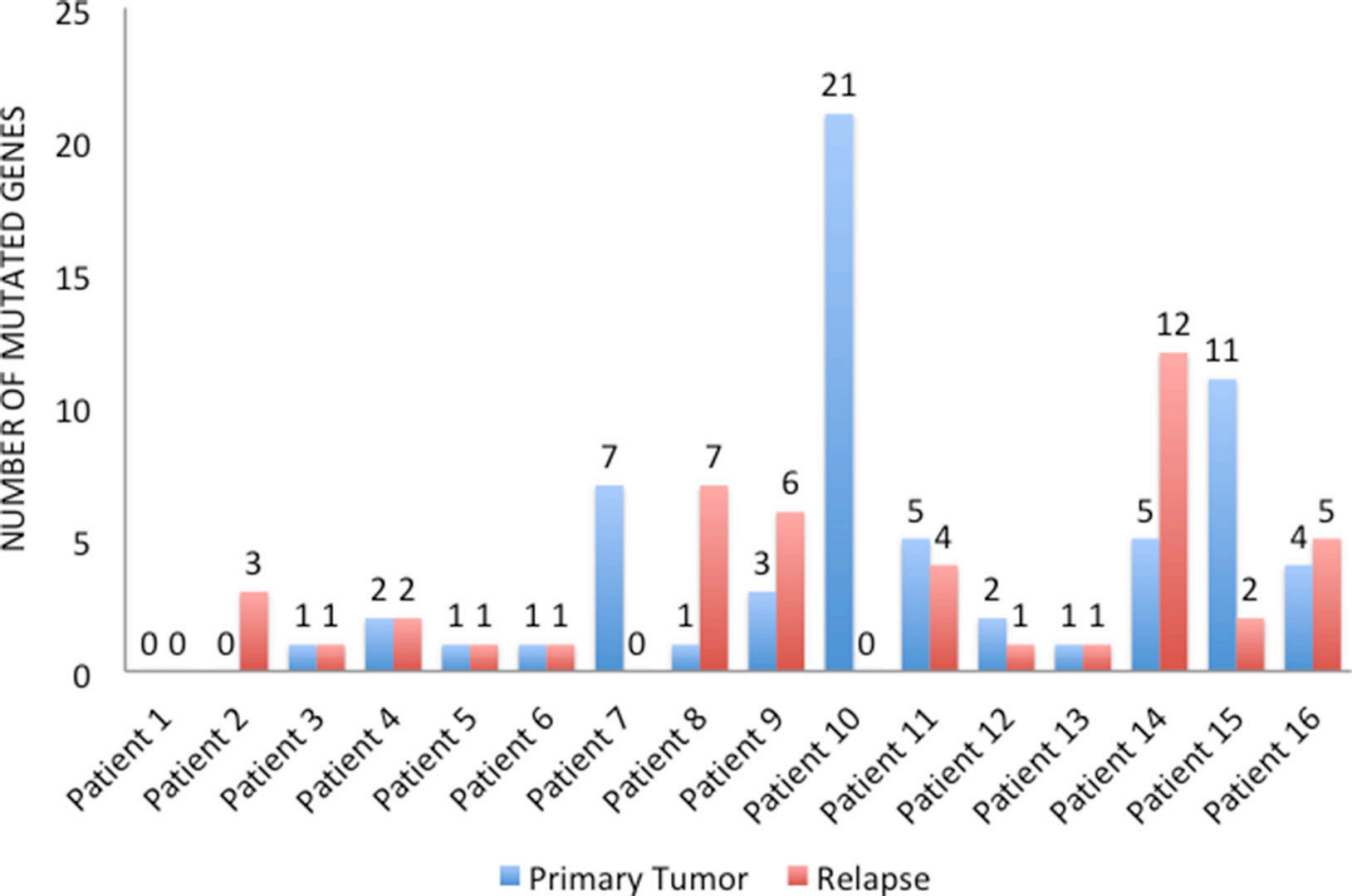
Number of mutated genes in primary tumors and relapsed tissues

**Figure 4:**
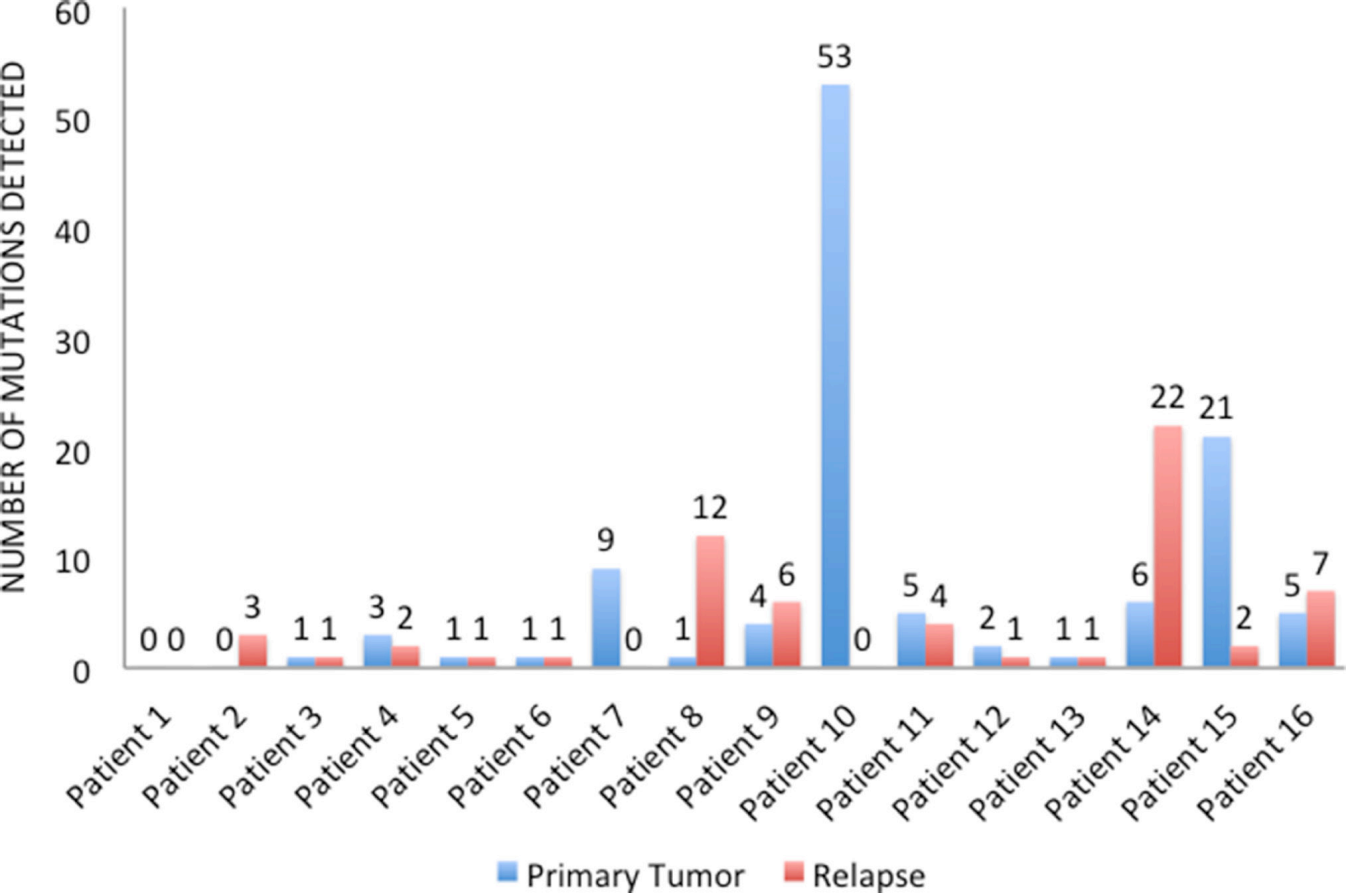
Number of pathogenic variants detected in primary tumors and relapsed tissues

In four cases (25%), primary tumors and relapsed tissues maintained the same mutational profile, whereas 12 (75%) patients changed pathogenic variants during progression. In particular, 9 patients (56.25%) acquired new mutations in the metastatic sites, while we find no primary tumor mutations in the relapsed tissues of 10 patients (62.5%). Overall, 5 patients (31.25%) showed more mutated genes (Figure 3) and more pathogenic variants (Figure 4) in the metastatic sites than in the primary tumor tissues. On the other hand, 5 patients (31.25%) had more mutated genes (Figure 3) and 6 (37.5%) had more pathogenic variants (Figure 4) in the primary tumor tissues.

**Figure 5:**
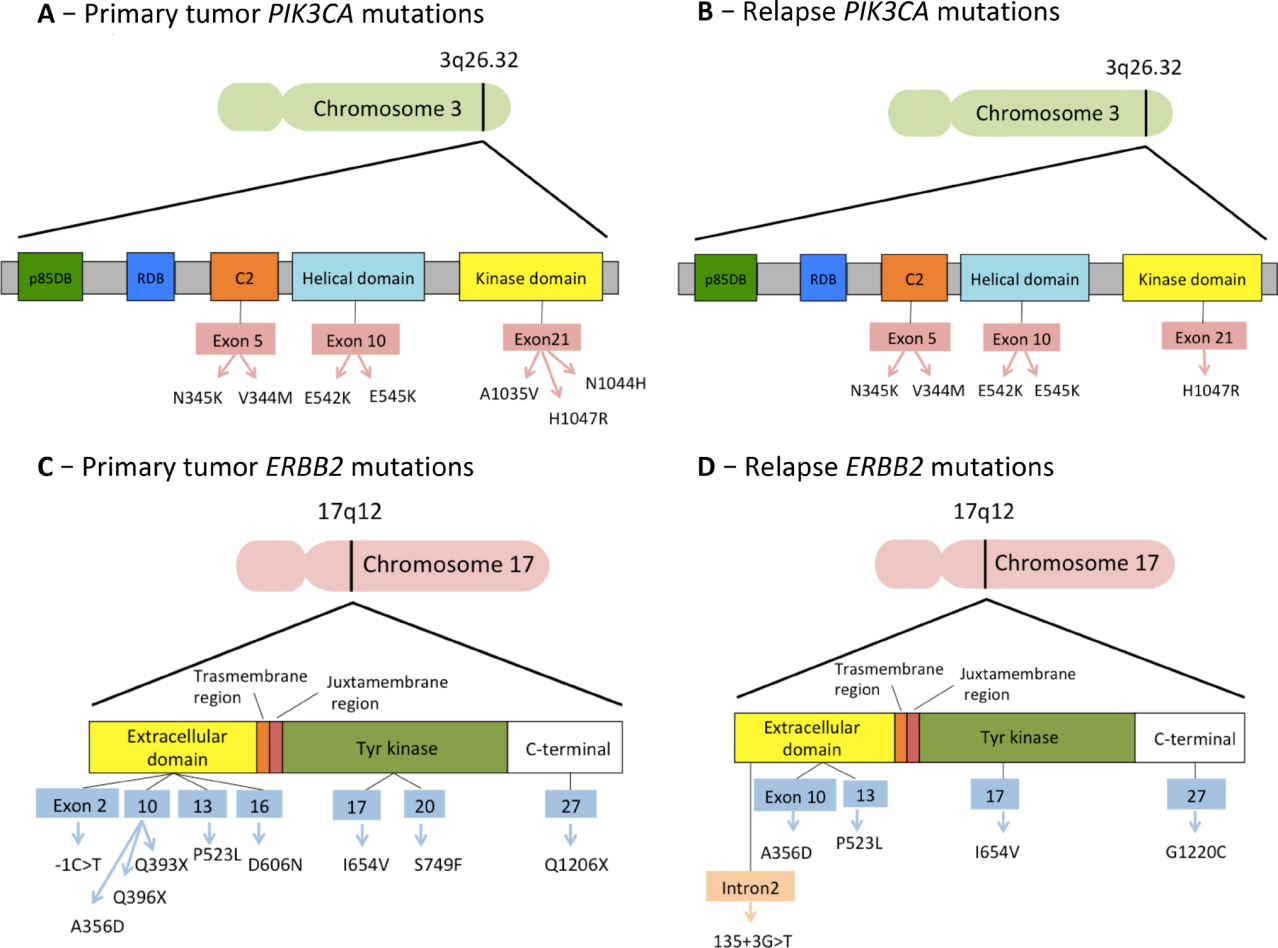
Mutations detected in the two most frequently mutated genes, *PIK3CA* and *ERBB2*

Finally, we analyzed the mutations detected in the genes that had already shown predictive value in clinical trials: *PIK3CA, ERBB2, ESR1* and *AKT1* genes (Table 2). Five out of 6 patients with a *PIK3CA* mutation in either primary tumor or relapse tissues exhibited one of the 3 “Hotspot” mutations shown to cause gains in protein enzymatic function and induce oncogenic transformation (E542 and E545 in the helical domain and H1047 in the kinase domain) [19, 20]. The other variants were N1044H, N345K, A1035V and V344M (Figure 5). With regard to the other genes, we found 11 different pathogenic or likely pathogenic variants for *ERBB2* (Figure 5), 5 different variants for *ESR1* and 4 different variants for *AKT1* (Table 2).

**Table 2:** Mutations detected in PIK3CA, ERBB2, ESR1 and AKT1 genes

	Primary tumor mutations				Relapse mutations			
	PIK3CA	ERBB2	ESR1	AKT1	PIK3CA	ERBB2	ESR1	AKT1
Patient 2	x	x	x	x	x	c.3658G>T, p.Gly1220Cys **(G3658T, G1220C)**	x	x
Patient 4	c.1624G>A, p.Glu542Lys (G1624A, E542K) c.3130A>C, p.Asn1044His **(A3130C, N1044H)**	x	x	x	c.1624G>A, p.Glu542Lys (G1624A, E542K)	x	x	x
Patient 5	c.3140A>G, p.His1047Arg (A3140G, H1047R)	x	x	x	c.3140A>G, p.His1047Arg (A3140G, H1047R)	x	x	x
Patient 6	x	c.1067C>A, p.Ala356Asp **(C1067A, A356D)**	x	x	x	c.1067C>A, p.Ala356Asp **(C1067A, A356D)**	x	x
Patient 7	x	c.-1C>T c.2246C>T, p.Ser749Phe **(C2246T, S749F)** c.1816G>A, p.Asp606Asn **(G1816A, D606N)**	x	x	x	x	x	x
Patient 8	x	x	x	x	x	x	c.382G>A, p.Val128Met **(G382A, V128M)**	c.117G>T, p.Lys39Asn **(G117T, K39N)** c.288-7C>T c.656C>T, p.Thr219Ile **(C656T, T219I)**
Patient 9	c.1624G>A, p.Glu542Lys (G1624A, E542K) c.1035T>A, p.Asn345Lys **(T1035A, N345K)**	x	x	x	c.1035T>A, p.Asn345Lys **(T1035A, N345K)**	x	x	c.46G>A, p.Gly16Arg **(G46A, G16R)**
Patient 10	c.3104C>T, p.Ala1035Val **(C3104T, A1035V)**	c.3526C>T, p.Gln1176Ter **(C3616T, Q1206X)** c.1096C>T, p.Gln366Ter **(C1186T, Q396X)** c.1087C>T, p.Gln363Ter **(C1177T, Q393X)**	x	c.32G>A, p.Trp11Ter **(G32A, W11X)**	x	x	x	x
Patient 11	c.1633G>A, p.Glu545Lys (G1633A, E545K)	c.1478C>T, p.Pro493Leu **(C1568T, P523L)**	x	x	c.1633G>A, p.Glu545Lys (G1633A, E545K)	c.1478C>T, p.Pro493Leu **(C1568T, P523L)**	x	x
Patient 12	x	x	x	x	c.3140A>G, p.His1047Arg (A3140G, H1047R)	x	x	x
Patient 14	x	x	c.1469T>G, p.Met490Arg **(T1469G, M490R)**	x	x	c.135+3G>T	c.600G>C,p.Trp200Cys **(G600C, W200C)** c.1048G>A, p.Ala350Thr **(G1048A, A350T)** c.1469T>G, p.Met490Arg **(T1469G, M490R)**	x
Patient 15	x	c.1870A>G, p.Ile624Val **(A1960G, I654V)**	x	x	x	c.1870A>G, p.Ile624Val **(A1960G, I654V)**	x	x
Patient 16	c.1030G>A, p.Val344Met **(G1030A, V344M)** c.3140A>G, p.His1047Arg (A3140G, H1047R)	x	x	x	c.1030G>A, p.Val344Met **(G1030A, V344M)** c.3140A>G, p.His1047Arg (A3140G, H1047R)	x	c.1370-26C>G	x

PIK3CA “Hotspot” mutations reported in red.

## DISCUSSION

Cancer is a dynamic and heterogeneous entity following the principles of clonal evolution, with different areas of the same primary tumor showing different genomic profiles and with metastases acquiring new molecular aberrations compared to their primary tumors [21–23]. Moreover, clonal heterogeneity, exacerbated by the selective pressures imposed by treatment during the life cycle of the disease, may confer greater resistance on anti-cancer treatments and radiation therapy [24]. Acquired drug resistance is common during the course of the disease. Therefore, there is an urgent need to monitor tumor evolution and ideally predict the onset of resistance to targeted therapies. In this context, emerging evidence from clinical trials assessing targeted therapies demonstrates that the genetic landscape of any given tumor will dictate its sensitivity or resistance profile to anticancer agents [25].

Against this backdrop, we evaluated a panel of 25 genes involved in targeted treatment resistance, comparing cancers sampled at first diagnosis (primary tumors) with their matched relapsed sites. Proper understanding of the mutational landscape of breast tumors through progression and metastatization should open new avenues for the assessment of resistance to therapy and the development of better targeted treatments, with a potential for improved clinical outcomes. The study allowed for the identification of the affected genes that were more prevalent in recurrences, compared with primary tumors, and that might be involved in the resistance to therapies.

In our study, we found an overall detection rate of mutations higher than that described in the literature. The most frequently mutated genes among the 25 analyzed were *PIK3CA* and *ERBB2* for both primary and relapsed tissues (Figure 5). Mutations of *PIK3CA* were present in 37.5% of primary tumors and relapsed tissues, while *ERBB2* mutations were present in 31.25% of our samples. These percentages are higher than those reported in previous research (30% for *PIK3CA* and 2–3% for *ERBB2*) [26, 27], indicating that our patients have been highly selected. It should be noted that only tumors with proved targeted treatment resistance, such as those relapsing or progressing under the targeted treatment, have been included in the study. This process increases the chance of finding the mutations responsible for that resistance. Other genes mutating with high frequency in our study were *mTOR* and *INPP4B*, which mutated in 31.25% of primary tumors, and *MAP2K2*, which mutated in 31.25% of relapsed sites. Overall, the most frequently mutated genes among those investigated are involved in the PI3K/AKT/mTOR and the MAPK signaling pathways. Conversely, mutations in genes involved in the cell-cycle regulation are not as crucial to the mechanism of treatment resistance.

In 75% of the patients, furthermore, the number and type of mutations changed with disease progression, as the immunohistochemical profile does in 50% of cases. In contrast with the previous literature, however, most of the time (68.75% of cases) the mutations detected did not increase in number throughout the natural history of the tumor. As already described, we included patients with targeted treatment resistant tumors. As a result, this might have contributed to selecting primary tumors with a high rate of mutations. Interestingly, 12 out of 14 patients (85.71%) with early-stage or locally advanced primary tumors showed a mutation in one of the genes analyzed. The high rate of mutation detected in primary BC tissues might explain the subsequent relapse and thus, it might justify a more targeted approach in neoadjuvant or adjuvant settings for patients exhibiting these mutations at diagnosis. It is noteworthy that previous studies indicated better prognoses for early BC patients harboring a *PIK3CA* mutation [28–30]. Along with the results shown by m-TOR and PI3K inhibitors in metastatic settings [31, 32], these data highlight a possible dual role of *PIK3CA* mutations in early versus advanced settings. In early HR-positive BC, *PIK3CA* may represent a predictive marker for benefit of endocrine therapies. In advanced HR-positive BC selected by primary endocrine therapy, *PIK3CA* contribute to endocrine resistance requiring combined endocrine and targeted therapy (i.e. everolimus or buparlisib). This is a research area in which more studies are clearly needed.

According to the results reported in recent clinical trials [6, 14, 15], mutations in *PIK3CA*, *ERBB2, AKT1* and *ESR1* genes are able to predict the response to some targeted therapies, and future research may find further associations. On these grounds, the mutations detected in the relapsed tissues/metastatic sites of 11 patients (68.75%) enrolled in our study could have guided subsequent treatment choices in a different way. More specifically, these patients underwent the standard treatments approved in that period for the metastatic setting. Nevertheless, 6 patients with *PIK3CA* mutations could have benefited from treatment with Everolimus, Buparlisib or Taselisib in their capacity as approved agents or in the context of clinical trials. Moreover, the patient with *AKT1* mutations could have benefited from Everolimus, while 3 patients with *ESR1* mutations could have benefited from Fulvestrant. Finally, 5 patients with *ERBB2* mutations could have benefited from treatment with Neratinib (Table 2).

In spite of such evidence, our study presents several limitations and must be considered hypothesis-generating. First of all, our study population is still small in sample size and the relapsed sites were biopsied at various time points throughout tumor course. Secondly, as patients were chosen based on sample availability, there may be inherent biases in patient selection. Furthermore, due to the retrospective nature of our analysis, samples were fixed and processed for storage in different periods and by different technicians, with no purpose of genomic analysis. This variability might have reduced the quality and preservation of some tissues, increasing the rate of variants detected in some of the samples. Overall, we described mutations with an allelic burden ≥3%. This was the case because, for research purposes, we wanted to describe the highest possible number of mutations present in our samples. Nevertheless, in some samples with a lower DNA quality we found a high number of variants, therefore we decided to use the cut-off of 5%. This is the detection limit of Sanger sequencing, the technique used in clinical practice for the confirmation of NGS variant calls. Future research should be conducted to clarify the predictive role of each mutation for each targeted therapy and define the cut-off level for test positivity, in order to select which patients would benefit most from that targeted treatment.

The re-characterization of recurrences was provided through invasive tumor biopsies, as the standard of practice. Obtaining samples of metastatic tissue is notably impractical and complicated by spatial heterogeneity and sampling bias. An attractive alternative to overcome the limitation of repeated tissue sampling is provided by peripheral blood samples as a ‘liquid biopsy’ through the analyses of CTCs, ctDNA or exosomes. Nevertheless, tumor heterogeneity, different sequencing techniques, spatial as well as temporal factors, and potential germline DNA contamination may cause low rates of concordance between liquid biopsies and tumor tissue. So far, therefore, both tissue and blood-based NGS are necessary to describe the complex biology of BC [33]. Finally, our study only focused on gene mutations, but the recent development of additional high information content assays focused on abnormalities in DNA methylation, microRNA expression and protein expression can provide further opportunities to better characterize the molecular architecture of BC.

The ability to perform NGS on small FFPE samples, such as core biopsies, creates the opportunity to comprehensively characterize cancer-relevant genes and personalize therapies. Overall, our study leads to the following conclusions:

1. Almost all patients had at least one alteration potentially targetable with approved or investigational therapeutics. This finding indicates that routine genomic profiling may be instrumental in individualized pathway-directed therapies.
2. Our study reveals substantial discordances between primary tumors and metastases, which stresses the need for analysis of metastatic tissue at recurrences. The analysis of recurrent tumors prior to selecting treatment may provide additional insights, as both gains and losses of targets are observed. Furthermore, the analysis could contribute to treatment selection. In this setting, the role of liquid biopsy instead of biopsy of the metastatic site should be further investigated.
3. Overall, the most frequently mutated genes among those investigated were involved in the PI3K/AKT/mTOR and the MAPK signaling pathway. It is likely that the deregulation of these pathways plays a crucial role in the mechanisms of targeted treatment resistance. Future research should be conducted to identify patients with a molecular profile predictive of treatment resistance, which makes them eligible for more intensive and personalized adjuvant treatments.
4. The extreme complexity of cancer biology necessitates analysis not only on a single level, as the novel targets for molecularly targeted therapy can be revealed at a DNA, RNA and protein level, or epigenetically.

Whether these alterations drive disease progression as well as survival, and therefore represent suitable therapeutic targets for the treatment of metastatic BC or for adjuvant therapy aimed at preventing a relapse warrants further study. However, our study demonstrates that mutation events may be present at diagnosis or arise during cancer treatment. Thus, profiling primary and metastatic tumor tissues may be a major step in defining optimal treatments for BC patients.

## MATERIALS AND METHODS

### Study design

Patients affected by metastatic hormone receptor (HR) positive and/or HER2 positive BC were eligible for the study. In order to be enrolled, patients had to have tissue samples taken from the primary tumor and at least one relapsed site stored in the archives of our Pathology Department. Using a next-generation sequencing (NGS) technology, the Ion Torrent Personalized Genome Machine (PGM) (Life Technologies, Guilford, CT, USA), we evaluated a panel of 25 genes involved in the mechanisms of endocrine and targeted treatment resistance. We analyzed formalin-fixed and paraffin-embedded (FFPE) tissues of primary BCs and the matched tissues taken from local or distant relapsed sites. Therefore, we compared the cancer genes found in cancers sampled at first diagnosis (primary tumors) with those found in relapsed cancers, after progression during treatment. The study was approved by our local research ethics committee.

### Patients and samples

**Figure 6:**
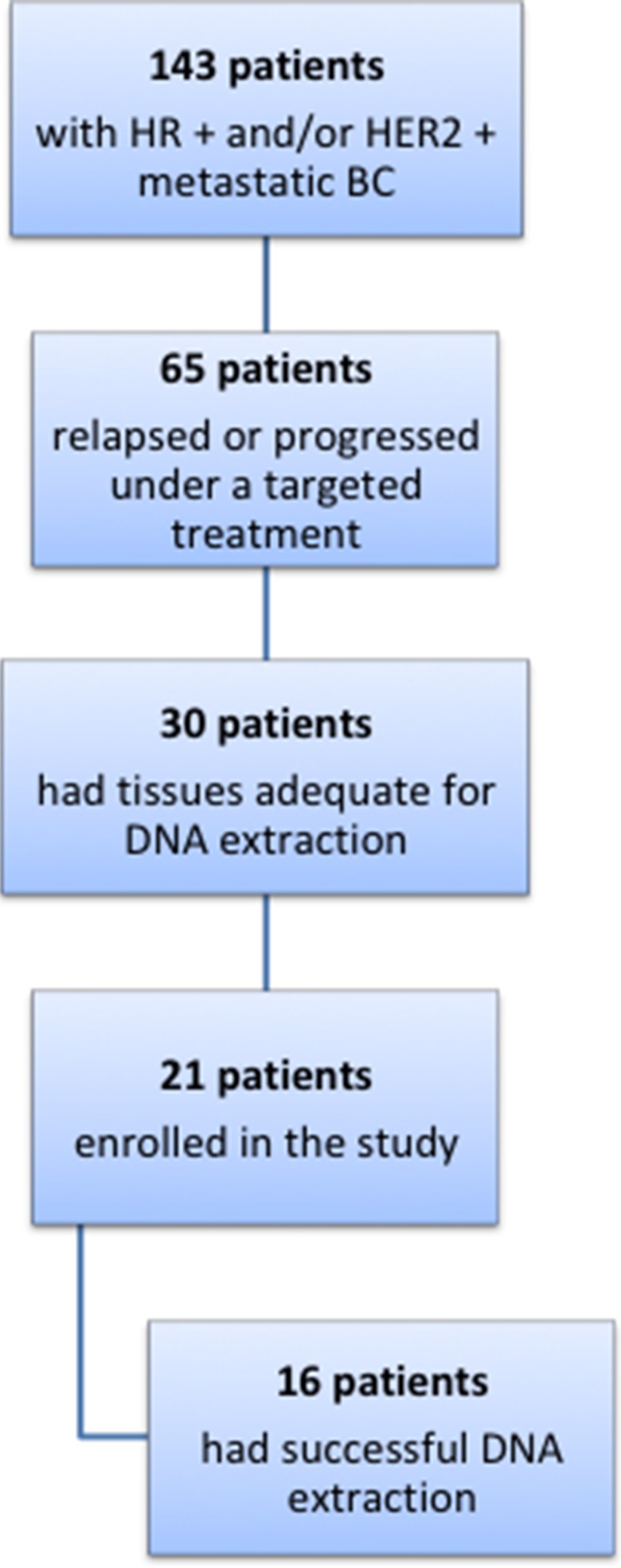
Flow diagram of patients evaluated for the study

We retrospectively identified 143 patients affected by metastatic hormone positive and/or HER2 positive BC, treated at the Modena Cancer Center in the last 20 years (Figure 6). Among these patients, 65 relapsed or developed disease progression during a targeted treatment, including endocrine therapy. Thirty of these patients had formalin-fixed and paraffin-embedded (FFPE) tissues taken from the primary tumor and from the local or distant recurrence stored in the archives of our Pathology Department. The FFPE samples were evaluated by a pathologist for specimen suitability, tumor quality and quantity, and selection of tumor areas for microdissection. Finally, only 21 patients gave consent to the use of their stored tissues for the study and were therefore included in the analyses.

DNA was purified from 6 to 8 5 μm-thin sections of each FFPE tissue using the Maxwell^®^ 16 FFPE Plus LEV DNA Purification Kit on the Maxwell^®^ 16 System (Promega). DNA sample quality and quantity were assessed through a Qubit 2.0 fluorometer and the Qubit™ dsDNA HS Assay Kit (ThermoFisher), in order not to overestimate the concentration of stressed FFPE-purified DNAs. In 16 out of 21 patients, the extraction was successful and the purified DNA was adequate for the sequencing.

Finally, DNA was successfully extracted from 32 FFPE blocks stored between 2000 and 2016. According to data reported by previous research, all of these FFPE blocks were considered suitable for DNA extraction [34]. In particular, 5 primary BC tissues were stored between 2000 and 2005, 8 specimens were stored between 2006 and 2010 and 3 specimens were stored between 2011 and 2016. Of secondary cancer tissues, only one was stored in 2004, 3 FFPE blocks were stored between 2006 and 2010, while 12 were stored between 2011 and 2016.

### The multi-gene panel

Twenty-five genes involved in the mechanisms of targeted treatment resistance were selected for the analyses. The list of genes and their role in treatment resistance are reported in Table 3 and described below:

**Table 3:** The gene panel

*EGFR*	*ERBB2*	*ERBB3*	*IGF1R*	*FGFR1*
*MET*	*AKT1*	*PDK1*	*mTOR*	*PIK3CA*
*PIK3R1*	*PTEN*	*INPP4B*	*BRAF*	*KRAS*
*MEK1*	*MEK2*	*ERK1*	*ERK2*	*CCDN1*
*CCDN2*	*CCDN3*	*CDK4*	*CDK6*	*ESR1*

- *EGFR*, *ERBB2* and *ERBB3*: this family of tyrosine kinase receptors is involved in cell proliferation control, differentiation and survival [35]. The mutation rate of ERBB2 in BC is about 2–3% [27].
- *IGF1R*, *FGFR1*, *MET*: in HR and/or HER2 positive BC, one of the possible mechanisms of resistance to endocrine and anti-HER2 agents is alternative signaling from other receptor tyrosine kinases [6].
- *AKT1, PDK1, mTOR, PIK3CA, PIK3R1, PTEN, INPP4B*: the PI3K/AKT/mTOR pathway is one of the main downstream pathways involved in cancer cell proliferation. Mutations of *PIK3CA* have been found in almost 30% of all sporadic BC [26] with a wide frequency range in BC subtypes [36, 37]. The majority of the mutations, “Hotspot” mutation, occurred in three sites: E542 and E545 in the helical domain, and H1047 in the kinase domain. These mutations cause gains in protein enzymatic function and induce oncogenic transformation [19, 20]. The somatic intragenic *PTEN* mutation frequency is <5% [38].
- *BRAF, KRAS, MEK1 (MAP2K1), MEK2 (MAP2K2), ERK1 (MAPK3), ERK2 (MAPK1):* the MAPK signaling pathway may lead to uncontrolled cell cycle, resistance to apoptosis and to chemotherapy, targeted therapies and radiotherapy [6].
- *CCDN1, CCDN2, CCDN3, CDK4, CDK6*: dysregulation of the cell-cycle machinery and activation of cyclin-dependent kinases (CDKs) represents one of the mechanisms of endocrine resistance. Following the literature, however, the mutation rate of these genes seems to be very low.
- *ESR1*: in HR positive BC, multiple mechanisms of endocrine resistance have been described, including mutations in the *ESR1* gene. Mutations in *ESR1* appear to be rare in treatment naive settings (up to 3% of primary tumors [39]) and more frequent in advanced BC (12.1% of a large cohort of advanced BC [40]).

### Study procedures

The Ion AmpliSeq™ technology (Thermo Fisher Scientific, Waltham, MA USA 02451) was used to design the gene panel described above. An optimized primer design for the FFPE samples encompassing the CDS and the UTR regions of the selected genes was created, in order to generate amplicons up to 175 bp. Libraries were prepared using the Ion AmpliSeq™ Library Kit 2.0 starting from 15 ng of gDNA/pool, according to the manufacturer's instructions. Template preparation was performed using an Ion OneTouch™ 2 System following the latest version of the manufacturer's manuals. The template positive Ion Sphere Particles (ISPs+) were sequenced on an Ion Torrent™ Personal Genome Machine^®^ (PGM™) System (Life Technologies Ltd, Paisley, UK) using the Ion 318™ Chip kit v2 following the Ion PGM™ Sequencing 200 Kit v2 manual. Run conditions were optimized to have at least 1000X coverage depth for 90% of target regions. In these conditions, LoD for mutation was about 1%. For the purpose of quality run assessment, we considered suitable for bioinformatics analysis only data coming from sequencing runs with the following run metrics: on-target % of reads >85%, amplicons with at least 1000 reads >90% and amplicon with no strand bias >95%. NGS sequence data were analyzed, processed and annotated as already described [41].

### Statistical analysis

Patient and tumor characteristics were retrieved from the electronic medical files of all patients. Data were summarized by frequency and percentage for categorical variables, and by median and range for continuous variables. The median disease-free survival (DFS) time for these patients was determined as the interval from surgery, or from the end of adjuvant chemotherapy, to any BC event including local, regional or distant recurrence or contralateral disease.

The bioinformatics analyses identified recurrently mutated genes in primary tumors and relapsed tissues. Using our NGS run conditions, we considered known pathogenic and likely pathogenic variants as reported in the literature so far, with an allelic burden ≥3%. For samples with lower DNA quality, we only considered pathogenic or likely pathogenic variants with an allelic burden (mutation frequency) ≥5%.
